# Association between Hemoglobin Levels and Diabetic Peripheral Neuropathy in Patients with Type 2 Diabetes: A Cross-Sectional Study Using Electronic Health Records

**DOI:** 10.1155/2017/2835981

**Published:** 2017-06-21

**Authors:** Jun Yang, Pi-jun Yan, Qin Wan, Hua Li

**Affiliations:** Department of Endocrinology, Affiliated Hospital of Southwest Medical University, Luzhou, Sichuan, China

## Abstract

**Objective:**

To investigate the relationship between hemoglobin levels and diabetic peripheral neuropathy (DPN) in type 2 diabetes mellitus (T2DM).

**Methods:**

1511 patients with T2DM were included in the study. DPN was diagnosed based on symptoms, signs, and laboratory tests. Hemoglobin was defined as both a continuous variable and a quartile category variable. We compared patient characteristics between the no diabetic peripheral neuropathy (NDPN) and DPN groups. Logistic regression was conducted to investigate the association of DPN with hemoglobin in all T2DM patients. Linear regression was also performed to investigate the impact of hemoglobin on the vibrating perception threshold (VPT).

**Results:**

Compared with the NDPN group, hemoglobin level in the DPN group was significantly lower (118.54 ± 16.91 versus 131.62 ± 18.32 g/L, *P* < 0.01). The prevalence of DPN increased by 50.1% (95% CI: 42.2–57.0%; *P* < 0.001) per standard deviation decrease in hemoglobin. Compared to the highest quartile of hemoglobin, the lower quartiles were associated with a significantly increased risk of DPN in the entire T2DM population (all *P* < 0.01). A per unit decrease in hemoglobin leads to a 0.12 (95% CI: 0.07–0.168) unit increase in VPT after adjustment for possible confounders (*P* < 0.001).

**Conclusions:**

Lower hemoglobin levels were associated with increased prevalence of DPN and higher VPT.

## 1. Introduction

It is estimated that there are 382 million people living with diabetes globally, and this number will rise to 592 million by 2035, as estimated by the International Diabetes Federation [1]. Individuals with diabetes are at increased risk of developing diabetic peripheral neuropathy (DPN)—a microvascular complication that may lead to diabetic foot disorders and even lower limb amputations. In a large cohort of people with DPN in the UK, 7% developed a diabetic foot wound after 1 year [2]. In a prospective study with long-term follow-up, the prevalence of DPN increased from 7.5% at baseline to 45% after 25 years [3]. In its early stages, DPN can be detected by vibrating perception threshold (VPT). Evidence has suggested that the VPT test appears to be an appropriate and reliable measure for screening DPN [4, 5].

The pathogenesis of DPN has not been clearly elucidated, although many common cardiovascular risk factors have been shown to be associated with DPN [6]. It is suspected that hemoglobin abnormalities may also be involved in the DPN pathogenesis through the process of bilirubin metabolism and erythrocyte deformability. As a byproduct of normal hemoglobin breakdown, bilirubin has antioxidative and anti-inflammatory properties and may effectively protect peripheral nerves against free-radical-induced damage [7–9]. Low levels of serum bilirubin seem to be associated with an increased risk of DPN. Another possible cause of DPN is the damaged deformability of red blood cells (RBCs) resulting from a decreasing level of hemoglobin. Hemoglobin plays a crucial role in the maintenance of erythrocyte deformability [10]. Decreased hemoglobin concentration can impair the ability of RBCs to change their shapes so as to adapt themselves to the tissue microenvironment. Impaired deformability has shown to be associated with microvascular complications in patients with diabetes, such as diabetic retinopathy and diabetic nephropathy [11–13].

With regard to DPN—another common microvascular complication of diabetes—however, there is, to our knowledge, very little evidence available on its association with hemoglobin. This study was conducted to explore the associations of hemoglobin with DPN and VPT.

## 2. Materials and Methods

### 2.1. Study Population

We screened records of a total of 3222 inpatients with type 2 diabetes mellitus (T2DM), who were consecutively referred to the Department of Endocrinology, the Affiliated Hospital of Southwest Medical University, Sichuan province, between August 2012 and June 2015. T2DM was diagnosed on the basis of oral glucose tolerance tests and the 1999 World Health Organization (WHO) criteria [14]. Eligible patients needed to meet the following criteria: (1) confirmed or newly diagnosed T2DM; (2) age ≥ 18 years; and (3) long-term residence (≥5 years) in the Sichuan province. Patients were excluded if they had (1) acute complications of diabetes including hyperosmolar coma, ketoacidosis, and hypoglycemic coma; (2) neuropathy resulting from acute and chronic inflammatory diseases, peripheral polyneuritis, infectious polyneuritis, vasculitis, and so on; (3) cervical spondylosis and lumbar spondylosis; or (4) malignancy and hematologic diseases. Moreover, we excluded patients who used medications that may affect the oxidant/antioxidant system, including vitamins (A, C, and E) and minerals (zinc and selenium) during the 6 months prior to study inclusion.

### 2.2. Diagnostic Criteria of DPN

Patients were diagnosed as having DPN in case of distal, symmetric, “glove and stocking” distributed loss of sensation, numbness, and prickling and if two or more of the following findings were presented: (1) VPT > 25 V; (2) 10 g Semmes-Weinstein monofilament test (SWMT) indicating weakening or loss of pressure sensation; (3) 40 g needle test indicating abnormal pricking sensation; and (4) weakening or loss of ankle and knee reflexes. Consequently, patients were divided into 2 groups: no diabetic peripheral neuropathy (NDPN) and DPN.

### 2.3. Clinical and Biochemical Measurements

We reviewed medical records of each eligible patient and used a structured form to extract information with regard to

1. hemoglobin (Hb), which was determined using an automated blood cell counter (Mindray BC-6800, Shenzhen, China);
2. VPT values, which were measured by a neurothesiometer (Bio-Thesiometer; Bio-Medical Instrument Co., Newbury, OH, USA). A VPT value > 25 V on either limb was considered abnormal;
3. demographic characteristics (gender and age);
4. medical history: menopausal status, diabetes duration, use of medications, coronary heart disease (CHD), cerebral infarction (CI), hypertension (HT), diabetic foot (DF), diabetic retinopathy (DR), diabetic nephropathy (DN), and peripheral vascular disease (PVD);
5. other clinical measurements: body weight, height, body mass index (BMI), blood pressure including both systolic blood pressure (SBP) and diastolic blood pressure (DBP), fasting blood glucose (FBG), glycated hemoglobin A1c (HbA1c), total cholesterol (TC), triglycerides (TG), high-density lipoprotein cholesterol (HDL-C), low-density lipoprotein cholesterol (LDL-C), serum creatinine (Cr), prothrombin time (PT), activated partial thrombin time (APTT), international normalized ratio (INR), fibrinogen, Hb, red blood cell distribution width (RDW), white blood count (WBC), neutrophil, lymphocyte count, neutrophil-to-lymphocyte ratio (NLR) urinary microalbumin, creatinine, urinary microalbumin-to-creatinine ratio (ACR; mg/g creatinine), estimated glomerular filtration rate (eGFR), and ankle-brachial index (ABI).

### 2.4. Statistical Analysis

Data were expressed as mean ± standard deviation (SD) for continuous variables, or as percentages (%) for categorical variables, unless otherwise specified. Differences in clinical and biochemical parameters between DPN and NDPN groups were compared using chi-square (*χ*^2^) tests for categorical variables and Mann–Whitney *U* test or one-way analysis of variance (ANOVA) depending on the assumption of normal distribution.

To explore whether lower Hb levels may be associated with DPN, binary logistic regression analysis was conducted. We included into the multivariable model only those baseline characteristics that seemed to be associated with the prevalence of DPN (indicated by *P* value < 0.1 on univariate analysis). Odds ratios (OR) and 95% confidence intervals (CI) were estimated. We then categorized patients into 4 quartile groups by the Hb level. Binary logistic regression analyses were conducted to investigate the association between quartiles of Hb and DPN, with adjustment for covariates. The highest quartile (Q1) served as the reference group, and OR and 95% CI were estimated. Possible dose-response relationships between Hb and DPN were examined by the trend test. Further, we compared VPT values across the 4 Hb quartile categories in all T2DM patients using ANOVA. Multiple stepwise linear regression analysis was conducted to identify possible risk factors of VPT.

All statistical analyses were conducted using SPSS 20.0 (SPSS, Chicago, IL, USA). All reported *P* values were two-sided. A *P* value < 0.05 was considered statistically significant.

### 2.5. Ethics Statements

The study conformed to the ethical guidelines of the 1975 Declaration of Helsinki and was approved by the human research ethics committee of the Affiliated Hospital of Southwest Medical University in Sichuan province. The requirement for informed consent was waived owing to the retrospective nature of the study.

## 3. Results

### 3.1. Baseline Characteristics of the Study Population

In total, 1511 T2DM patients (743 males and 768 females) were finally enrolled in this study. Baseline characteristics of all participants are shown in Table 1. Compared with patients in the NDPN group, patients in the DPN group were significantly older and had lower BMI and longer duration of diabetes (all *P* < 0.01). Moreover, patients in the DPN group showed greater prevalence of CHD, CI, HT, PVD, DF, DR, and DN than those in the NDPN group. In this group, SBP, PP, FBG, HbA1c, Cr, neutrophil count, WBC count, PT, APTT, INR, fibrinogen, VPT, and urinary ACR levels were elevated, whereas DBP, TC, TG, lymphocyte count, Hb, ABI, and eGFR were decreased (*P* < 0.01 or *P* < 0.05). No between-group differences in HDL-C, LDL-C, RDW, and gender were detected (all *P* > 0.05).

### 3.2. Association between Hb and DPN in T2DM Patients

The prevalence of DPN was increased by 50.1% (95% CI: 42.2–57.0%; *P* value < 0.001) per standard deviation decrease in Hb. Such an association was stronger for male T2DM patients (OR = 1.618, 95% CI: 1.527–1.691, *P* value < 0.001) than in female T2DM patients (OR = 1.388, 95% CI: 1.257–1.495, *P* value < 0.001). Compared to the highest quartile of Hb, the lower quartiles were associated with a significantly increased risk of DPN in the entire T2DM population (all *P* values < 0.01).

### 3.3. Association between Quartiles of Hb and DPN in Male and Female T2DM Patients

Binary logistic regression analysis indicated that, when compared to the highest quartile of Hb (Q4), the lower quartiles (Q3, Q2, and Q1) were associated with a significantly increased risk of DPN in the entire T2DM study population (all *P* values < 0.01). However, only the lowest quartile of Hb was associated with a significantly increased risk of DPN among female T2DM study subjects. There was a significant upward trend in the prevalence of DPN per quartile decrease in Hb, in the study population, regardless of gender (Table 2).

### 3.4. Association between VPT Values and Quartiles of Hb Levels in All T2DM Patients

The VPT values decreased per quartile increase in Hb quartiles in all T2DM patients (all *P* < 0.01; Table 3) in this study. A lower quartile of Hb level (Q3, Q2, and Q1) was associated with a higher value of VPT. Per unit decrease in Hb resulted in a 0.12 (95% CI: 0.07–0.168) increase in the value of VPT (Table 4).

## 4. Discussion

In this large retrospective sample of patients with T2DM in China, lower Hb levels were associated with an increased risk of DPN and a higher value of VPT. Per standard deviation, decrease in Hb was associated with an increase in the prevalence of DPN by 50.1% (95% CI: 42.2–57.0%). Compared with participants who were categorized to the highest quartile of Hb level (Q4), those with a lower quartile of Hb level (Q3, Q2, and Q1) showed a 2.4-, 5.0-, and 8.8-fold increase in the prevalence of DPN, respectively. This association was stronger among males than females, and a per unit decrease in Hb resulted in a 0.12 increase in VPT value.

The relationship between Hb and DPN observed in this study was consistent with our study hypothesis. Hb is known as a protein in RBCs that helps store and transport oxygen. Reduction in Hb will impair oxygen transport to the peripheral nervous system. Neurovascular factors causing damage to blood vessels that carry oxygen and nutrients to nerves can contribute to DPN progression [15]. This situation worsens in cases with low Hb levels. Furthermore, Hb plays a major role in erythrocyte deformability, whereby oxygen transfer can be facilitated and flow resistance can be reduced under shear stress. Decreased cytoplasmic Hb concentration in erythrocytes can reduce cytoplasmic viscosity and impair erythrocyte deformability, with resultant RBC-induced damage to the endothelium within the microcirculation; this leads to endothelial dysfunction and diabetic microvascular complications [16]. Despite limited clinical evidence correlating DPN to Hb or erythrocyte deformability, previous cross-sectional studies have investigated the association of erythrocyte deformability with diabetic nephropathy and retinopathy and indicated that impairment in erythrocyte deformability is associated with loss of renal function and the risk of diabetic retinopathy in patients with diabetes [11–13].

Another possible mechanism underlying the association between Hb and increased risk of DPN is the reduced production of bilirubin, a byproduct of normal hemoglobin breakdown with antioxidant and anti-inflammatory effects on neural tissue. An association between serum total bilirubin and the morphology of corneal nerve fibers was found in Japanese patients with uncontrolled T2DM [17]. Several cross-sectional studies have, moreover, consistently suggested a low level of serum total bilirubin may be an independent risk factor of DPN in T2DM [18–21].

Interestingly, we found that the inverse association of DPN with Hb was stronger in males than in females. A similar interaction by gender has not been reported in previous studies investigating associations between erythrocyte deformability or bilirubin and microvascular complications in diabetes [11–13, 17–21]. It is acknowledged that men have a mean Hb level that is approximately 12% higher than in women [22]. Therefore, we suspect men are likely more sensitive to decrease in Hb after having had long-term consistently greater levels of Hb. Although previous research showed that males have a higher frequency of diabetic neuropathy [23], more severe symptoms related to diabetic neuropathy [24], and earlier onset of DPN [25], our study suggested that even gender may modify the association between Hb and DPN.

The inverse association between Hb and VPT was found to be consistent, with increased risk of DPN associated with lower Hb levels. It seems that VPT has high sensitivity and specificity to screen DPN [26]. Among 6487 patients, the prevalence of large-fiber neuropathy detected by VPT (≥15 V) was 21% in those with diabetes duration < 5 years and 37% in those with diabetes duration of >10 years [27]. Our findings indicated that large nerve fibers may be vulnerable to low levels of Hb, and this impairment seems to occur in the early stage of DPN.

To the best of our knowledge, this is the first study conducted to explore the association between Hb and DPN in patients with T2DM. Another strength of this study is the inclusion of a large number of patients, allowing for a high power to detect a possible association. Besides, we have carefully controlled for established and potential risk factors for DPN and VPT.

Some limitations in this retrospective cohort study need to be considered. First, results from electromyography, which would help discriminate the specific peripheral nerves involved, were not available for analysis in our study. Therefore, we cannot determine whether the DPN is a predominantly sensory, motor, or combined neuropathy. Second, our analysis was based on the information routinely collected in electronic health records. When using this kind of data source, problems with inaccuracy and incompleteness of the information cannot be neglected. Issues of misclassification may lead to biased estimates. Residual confounding by other unmeasured factors, such as dietary habits and lifestyle behavior, is still possible. However, we have adjusted for all the known confounders that could be observed from our electronic health records. Third, as a cross-sectional study, our study could not confirm the causal effects of Hb on the development of DPN; a reverse causal association cannot be precluded. However, the positive associations of DPN and VPT with Hb observed in our study may help generate a hypothesis that abnormal Hb may be involved in the pathogenesis of DPN. Fourth, we only included eligible patients from a single hospital. Therefore, findings from this study may be generalizable to other populations only with considerable caution.

In conclusion, this large retrospective study in a cohort of patients with T2DM suggested that a lower level of Hb was associated with an increased risk of DPN and abnormal VPT. Particular attention in clinical practice may be necessary in diabetic patients with low Hb levels for DPN prevention. Given its cross-sectional design, this study alone cannot possibly establish a causal relationship between Hb levels and DPN and confirm all the observed associations.

## Figures and Tables

**Table 1:** Baseline characteristics of patients in NDPN and DPN groups.

Characteristics	NDPN (*n* = 1287)	DPN (*n* = 224)	*P* value
Male/female	626/661	117/107	0.321
Diabetes duration (years)	7.20 ± 6.15	10.31 ± 7.48	<0.001
Age (years)	58.61 ± 11.16	66.58 ± 9.92	<0.001
BMI (kg/m^2^)	24.39 ± 3.68	23.50 ± 3.97	<0.001
SBP (mmHg)	132.06 ± 20.54	134.72 ± 22.25	0.036
DBP (mmHg)	72.44 ± 12.01	69.75 ± 12.69	0.002
PP (mmHg)	59.64 ± 18.35	64.97 ± 19.44	<0.001
TC (mmol/L)	4.90 ± 1.36	4.63 ± 1.33	0.003
TG (mmol/L)	2.45 ± 2.78	1.88 ± 1.42	<0.001
HDL-C (mmol/L)	1.17 ± 0.37	1.16 ± 0.33	0.580
LDL-C (mmol/L)	2.78 ± 1.01	2.75 ± 0.99	0.438
FBG (mmol/L)	10.68 ± 5.05	11.78 ± 5.88	0.005
HbA1c (%)	9.40 ± 2.48	10.10 ± 2.67	<0.001
Hb (g/L)	131.62 ± 18.32	118.54 ± 16.91	<0.001
RDW (%)	13.14 ± 1.22	13.25 ± 1.30	0.417
WBC (^∗^×10^12^/L)	6.71 ± 2.35	7.43 ± 2.98	<0.001
Neutrophil count (^∗^×10^9^/L)	4.49 ± 2.22	5.40 ± 2.83	<0.001
Lymphocyte count (^∗^×10^9^/L)	1.69 ± 0.68	1.44 ± 0.58	<0.001
NLR	3.15 ± 2.70	4.58 ± 4.23	<0.001
PT	12.50 ± 1.18	13.10 ± 1.96	<0.001
APTT	31.36 ± 5.79	32.94 ± 8.65	0.031
INR	1.03 ± 0.08	1.07 ± 0.18	0.002
Fibrinogen (mmol/L)	3.54 ± 1.23	4.45 ± 1.63	<0.001
Urinary ACR (mg/g)	212.53 ± 23.76	432.12 ± 71.36	<0.001
Cr (*μ*mol/L)	71.11 ± 45.42	89.91 ± 58.10	<0.001
eGFR (mL/min/1.73 m^2^)	94.28 ± 25.05	77.64 ± 28.01	<0.001
ABI	1.04 ± 0.13	0.94 ± 0.24	<0.001
VPT (V)	12.98 ± 4.75	36.30 ± 8.91	<0.001
VPT >25 V (%)	0 (0)	224 (100)	<0.001
Abnormal pricking sensation	662 (51.44)	151 (67.41)	<0.001
Abnormal nylon monofilament	0 (0)	20 (8.93)	<0.001
Two abnormal DPN screening	0 (0)	151 (67.41)	<0.001
Three abnormal DPN screening	0 (0)	16 (7.14)	<0.001
CHD (%)	100 (7.77)	38 (16.96)	<0.001
CI (%)	232 (18.03)	72 (32.14)	<0.001
HT (%)	650 (50.51)	144 (64.29)	<0.001
DF (%)	61 (4.74)	53 (23.66)	<0.001
PVD (%)	90 (6.99)	63 (28.13)	<0.001
DR (%)	145 (11.27)	48 (21.43)	<0.001
DN (%)	427 (33.18)	118 (52.68)	<0.001

Note: DPN: diabetic peripheral neuropathy; NDPN: no diabetic peripheral neuropathy; BMI: body mass index; SBP: systolic blood pressure; DBP: diastolic blood pressure; PP: pulse pressure; TC: total cholesterol; TG: triglyceride; HDL-C: high-density lipoprotein cholesterol; LDL-C: low-density lipoprotein cholesterol; FBG: fasting blood glucose; HbA1c: glycated hemoglobin A1c; Hb: hemoglobin; RDW: red blood cell distribution width; WBC: white blood cell; NLR: neutrophil to lymphocyte ratio; PT: prothrombin time; APTT: activated partial thrombin time; INR: international normalized ratio; ACR: microalbumin to creatinine ratio; Cr: creatinine; eGFR: estimated glomerular filtration rate; ABI: ankle-brachial index; VPT: vibration perception threshold; CHD: coronary heart disease; CI: cerebral infarction; HT: hypertension; DF: diabetic foot; PVD: peripheral vascular disease; DR: diabetic retinopathy; DN: diabetic nephropathy; continuous variables were presented as mean ± SD and categorical variables as percentage; ^∗^*P* < 0.05; *P* < 0.01 compared with NDPN group.

**Table 2:** Association between quartiles of Hb with DPN in different groups of T2DM patients.

Hb (g/L)	All T2DM (*n* = 1511)	*P* value	Male T2DM (*n* = 743)	*P* value	Female T2DM (*n* = 768)	*P* value
Per SD decrease	1.501 (1.422–1.570)	<0.001	1.618 (1.527–1.691)	<0.001	1.388 (1.257–1.495)	<0.001
Quartiles of Hb						
Q1	8.820 (4.932–15.774)	<0.001	35.289 (10.846–114.818)	<0.001	2.665 (1.490–4.767)	0.001
Q2	5.003 (2.762–9.065)	<0.001	14.3 (4.399–46.483)	<0.001	1.440 (0.795–2.609)	0.228
Q3	2.475 (1.311–4.672)	0.005	3.56 (0.976–12.988)	0.001	0.903 (0.437–1.867)	0.784
Q4	1 (reference)		1 (reference)		1 (reference)	
Q1, Q2, Q3 versus Q4	1.978 (1.703–2.298)	<0.001	3.246 (2.497–4.219)	<0.001	1.552 (1.275–1.890)	<0.001

Hb: hemoglobin; SD: standard deviation; Q: quartile.

**Table 3:** Comparison across quartiles of Hb in all T2DM patients.

Item		Hb quintiles			*P* value for trend
	Q1	Q2	Q3	Q4	
VPT (v)	20.64 ± 11.93	17.30 ± 10.35^∗^	15.02 ± 8.21^∗^	12.49 ± 6.63^∗^^#▲^	<0.001

Versus Q1, ^∗^*P* value < 0.01; versus Q2, ^#^*P* value < 0.01; versus Q3, ^▲^*P* value < 0.01. Hb: hemoglobin; Q: quartile; VPT: vibration perception threshold.

**Table 4:** Multiple stepwise regression analysis of VPT value in all T2DM patients.

Variable	Unstandardized Beta	95% CI	*P* value
Age	0.252	0.175 to 0.329	<0.001
Gender	−3.801	−5.490 to −2.112	<0.001
HbA1c	0.552	0.241 to 0.863	0.001
Hb	−0.119	−0.168 to −0.070	<0.001
Fibrinogen	1.403	0.762 to 2.044	<0.001
ABI	−7.633	−12.276 to −2.990	0.001

HbA1c: glycated hemoglobin A1c; Hb: hemoglobin; ABI: ankle-brachial index.
